# Roles of ESCRT Proteins ALIX and CHMP4A and Their Interplay with Interferon-Stimulated Gene 15 during Tick-Borne Flavivirus Infection

**DOI:** 10.1128/JVI.01624-21

**Published:** 2022-02-09

**Authors:** Pham-Tue-Hung Tran, Abhilash I. Chiramel, Magnus Johansson, Wessam Melik

**Affiliations:** a School of Medical Sciences, Inflammatory Response and Infection Susceptibility Centre (iRiSC), Örebro University, Örebro, Sweden; b Innate Immunity and Pathogenesis Section, Laboratory of Virology, Rocky Mountain Laboratories (RML), National Institute of Allergy and Infectious Diseases (NIAID), National Institutes of Health (NIH), Hamilton, Montana, USA; Cornell University

**Keywords:** tick-borne flaviviruses, ESCRT, TSG101, ALIX, CHMP4A, ISG15, HERC5, virus late domain, replicons, replication, assembly, NS3, envelope

## Abstract

Flaviviruses are usually transmitted to humans via mosquito or tick bites. During infection, virus replication and assembly, whose cellular sites are relatively close, are controlled by virus proteins and a diverse range of host proteins. By siRNA-mediated gene silencing, we showed that ALIX and CHMP4A, two members of the host endosomal sorting complex required for transport (ESCRT) protein machinery, are required during flavivirus infection. Using cell lines expressing subgenomic replicons and replicon virus-like particles, we demonstrated specific roles for ALIX and CHMP4A in viral replication and assembly, respectively. Employing biochemical and imaging methodology, we showed that the ESCRT proteins are recruited by a putative specific late (L) domain motif LYXLA within the NS3 protein of tick-borne flaviviruses. Furthermore, to counteract the recruitment of ESCRT proteins, the host cells may elicit defense mechanisms. We found that ectopic expression of the interferon-stimulated gene 15 (ISG15) or the E3 ISG15-protein ligase (HERC5) reduced virus replication by suppressing the positive effects of ALIX and CHMP4A. Collectively, these results have provided new insights into flavivirus-host cell interactions that function as checkpoints, including the NS3 and the ESCRT proteins, the ISG15 and the ESCRT proteins, at essential stages of the virus life cycle.

**IMPORTANCE** Flaviviruses are important zoonotic viruses with high fatality rates worldwide. Here, we report that during infection, the virus employs members of ESCRT proteins for virus replication and assembly. Among the ESCRT proteins, ALIX acts during virus replication, while CHMP4A is required during virus assembly. Another important ESCRT protein, TSG101, is not required for virus production. The ESCRT, complex, ALIX-CHMP4A, is recruited to NS3 through their interactions with the putative L domain motif of NS3, while CHMP4A is recruited to E. In addition, we demonstrate the antiviral mechanism of ISG15 and HERC5, which degrades ALIX and CHIMP4A, indirectly targets virus infection. In summary, we reveal host-dependency factors supporting flavivirus infection, but these factors may also be targeted by antiviral host effector mechanisms.

## INTRODUCTION

The genus *Flavivirus* includes highly pathogenic viruses that cause encephalitic or hemorrhagic diseases in humans. Many flaviviruses are transmitted by mosquitoes, including West Nile virus (WNV), Japanese encephalitis virus (JEV), yellow fever virus (YFV), and Zika virus (ZIKV). The serocomplex of tick-borne flaviviruses (TBFV) include tick-borne encephalitis virus (TBEV), Powassan virus (POWV), Kayasanur forest disease virus (KFDV), and the naturally attenuated Langat virus (LGTV) (1), which is often used as the TBFV model.

Flaviviruses are enveloped viruses with a positive-sense single-stranded RNA genome that encodes a single polyprotein. Cleavages of the polyprotein by host and viral proteases result in 10 functional viral proteins. These include the following structural proteins: capsid (C), premembrane (prM), and envelope (E) proteins for virus encapsidation-envelopment, and seven nonstructural (NS) proteins (NS1 to NS5) essential for viral RNA replication (2–6) and assembly (7–9). Among the NS proteins, NS3 with its cofactor NS2B functions as a protease for the polyprotein cleavage (10–12). Furthermore, the C-terminal domain of NS3 possesses an ATPase activity for capping of viral RNA and a helicase activity for separation of the intermediate double-stranded RNA formed during virus replication (13). In addition to these enzymatic roles, NS3 also regulates virus assembly (9) by interacting with prM-E, NS2A, and the viral genomic RNA (7, 8).

During infection, flavivirus proteins coordinate to remodel the endoplasmic reticulum (ER), resulting in invaginated replication vesicles, which scaffold the replication complex of the NS proteins and viral genomic RNA (6, 14, 15). Flavivirus replication is coupled with virus assembly at the ER (6), followed by virus egression from the ER through the Golgi. During the transit, prM is cleaved by a host furin-like protease to form the M protein, which results in maturation of infectious virions (16, 17). Mature virus particles are secreted by host cells via exocytosis (16).

In eukaryotic cells, the endosomal sorting complex required for transport (ESCRT) machinery is a conserved membrane remodeling complex, which plays essential roles in generation of the multivesicular body, membrane abscission during mitosis, nuclear envelope repairing, and autophagosome closure (18). During formation of the ESCRT complex, components of the early ESCRT proteins: ALG-2-interacting protein X (ALIX), ESCRT-0, ESCRT-I, or ESCRT-II proteins are recruited to ubiquitinated proteins or to proteins containing the late (L) domain sequences (PPXY, PT/SAP, and YXXL). This is followed by the attachment of ESCRT-III proteins to elicit membrane scission (18).

During retrovirus, influenza, and filovirus infection, members of the ESCRT machinery – including ALIX, the ESCRT-I tumor susceptibility 101 protein (TSG101), and the ESCRT-III charged multivesicular body protein 4 (CHMP4) – are essential for viral budding and egress (19–22). In the context of the mosquito-borne flaviviruses (MBFV), TSG101 and CHMP4 proteins are essential for JEV and DENV virion production (23), and the ALIX protein is employed by YFV (24). However, no studies of these ESCRT proteins have previously been conducted in relation to the TBFV.

On sensing viral RNA, cells produce interferon to upregulate the expression of interferon-stimulated genes (ISGs) to establish an antiviral state (25). ISGs can directly target viral products for degradation or act on pathways usurped by viruses to limit virus infection (25). Interferon-stimulated gene product 15 (ISG15), which is a ubiquitin-like protein, can be conjugated to a variety of host proteins by the E3 protein ligase HERC5 (26, 27) in a process called ISGylation. During influenza A virus (IAV) infection, ISGylation of TSG101 blocks post-Golgi trafficking of hemagglutinin (HA) to the plasma membrane, which restricts virus budding (20). In the context of MBFV, infection results in the recruitment of the ESCRT proteins TSG101, CHMP2, and CHMP4 to orchestrate virus assembly at the ER (23), but how ISGylation precisely influences ESCRT recruitment in flavivirus infection is not understood.

Given the essential roles of ESCRT proteins and ISG15, we characterized the functions of ESCRT proteins, including TSG101, ALIX, and CHMP4A; the roles of HERC5 and ISG15; during TBFV infection using an array of TBFV (LGTV, TBEV_Sofjin_, KFDV, and POWV) and compared these with MBFV (WNV Kunjin strain [WNV_Kunjin_] and ZIKV). We showed that the ESCRT proteins were recruited by the virus NS3 and E proteins of the TBFV. Furthermore, ectopic expression of ISG15 or HERC5 inhibited TBFV replication by degradation of the ESCRT proteins. Thus, this work reveals the use of ESCRT proteins during flavivirus infection as well as a potential antiviral defense mechanism of the host.

## RESULTS

### ESCRT proteins ALIX and CHMP4A are essential for virus production.

**FIG 1:**
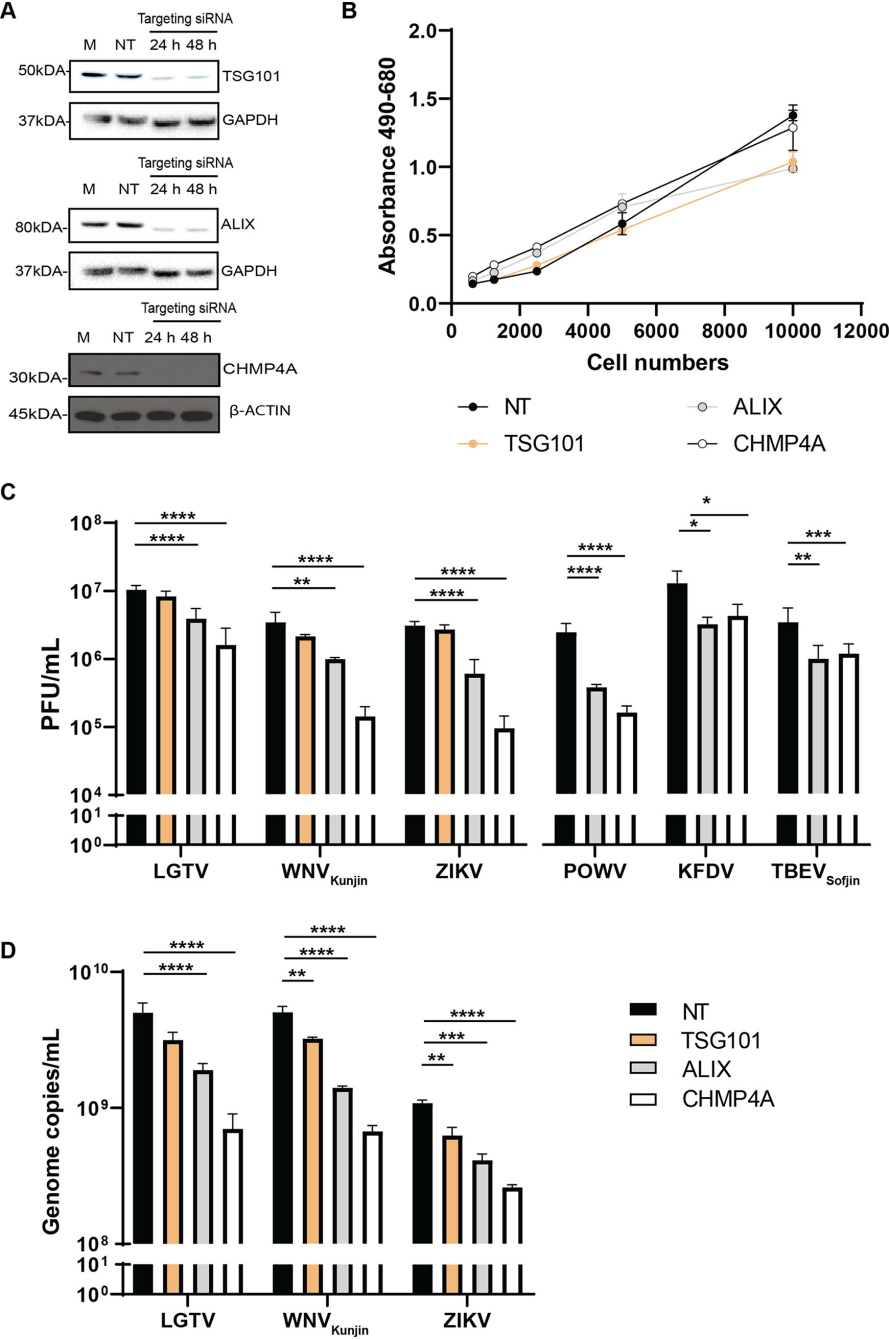
ESCRT proteins ALIX and CHMP4A are essential for virus production. (A) Immunoblotting of A549 cell lysates was performed 24–48 h posttransfection with the antibody against either CHMP4A, TSG101, or ALIX after specific siRNA treatments. The expression levels were normalized using the endogenous proteins, either glyceraldehyde-3-phosphate dehydrogenase (GAPDH) or β-actin. (B) Cytotoxicity assay was performed by measuring the amount of lactate dehydrogenase (LDH) enzyme released into cell culture media during siRNA treatments. When the plasma membrane is damaged, the endogenous LDH is released into media and reacts to substrates from the assay, generating a red formazan product that can be measured spectrophotometrically at 490 nm and normalized to the absorbance at 680 nm. (C) The virus titers in supernatants from the TSG101, ALIX, or CHMP4A siRNA-treated cells were compared to the NT siRNA-treated cells after Langat virus (LGTV), West Nile virus strain Kunjin (WNV_Kunjin_), or Zika virus (ZIKV), Powassan virus (POWV), Kyasanur forest disease virus (KFDV), and tick-borne encephalitis virus strain Sofjin (TBEV_Sofjin_) infection. The virus titers were measured by plaque assay. (D) The virus genome copy numbers were measured by qPCR from supernatants of the indicated siRNA-treated cells. The experiments were conducted independently three times with two technical repeats. The *P values* are indicated using * *P* < 0.05, ** *P* < 0.01, *** *P* < 0.001, and **** *P* < 0.0001.

As ESCRT proteins play specific roles during retrovirus (18) and MBFV infection (23, 24), we characterized the effects of the three essential ESCRT proteins—TSG101, ALIX, and CHMP4A—during TBFV infection. We initially silenced expression of these genes by transfecting A549 cells with targeting siRNA. Immunoblotting of cell lysates revealed that gene knockdown reduced protein expression (Fig. 1A). Furthermore, siRNA treatments that targeted these ESCRT expressions did not significantly increase cytotoxicity monitored by the amount of lactate dehydrogenase (LDH) enzyme released into cell culture media (Fig. 1B).

Subsequently, cells were infected with TBFV, including POWV, KFDV, TBEV_Sofjin_, and LGTV, and were compared to 2 MBFV – WNV_Kunjin_ and ZIKV. The virus titers in cell culture supernatants were then enumerated by plaque assay (Fig. 1C). Interestingly, silencing of ALIX and CHMP4A resulted in reduction of extracellular virus titers between a 0.5 and 1 logarithm (log), compared to the nontargeting (NT) siRNA control (Fig. 1C). Surprisingly, depletion of TSG101 showed modest effects on the viruses investigated, compared to the NT siRNA control (Fig. 1C).

We further examined genome copy numbers of LGTV, WNV_Kunjin_, and ZIKV in virus-infected cell culture supernatants by qPCR. In accordance with the virus titers, depletion of either ALIX or CHMP4A resulted in significant reduction of genome copy numbers, whereas depletion of TSG101 had significant but very limited effects on the indicated viruses (Fig. 1D). Overall, these data suggest that ALIX and CHMP4A are essential for flavivirus production and may facilitate virus RNA replication.

### Reduction of intracellular virus particles and genome copy numbers during ALIX or CHMP4A depletion.

**FIG 2:**
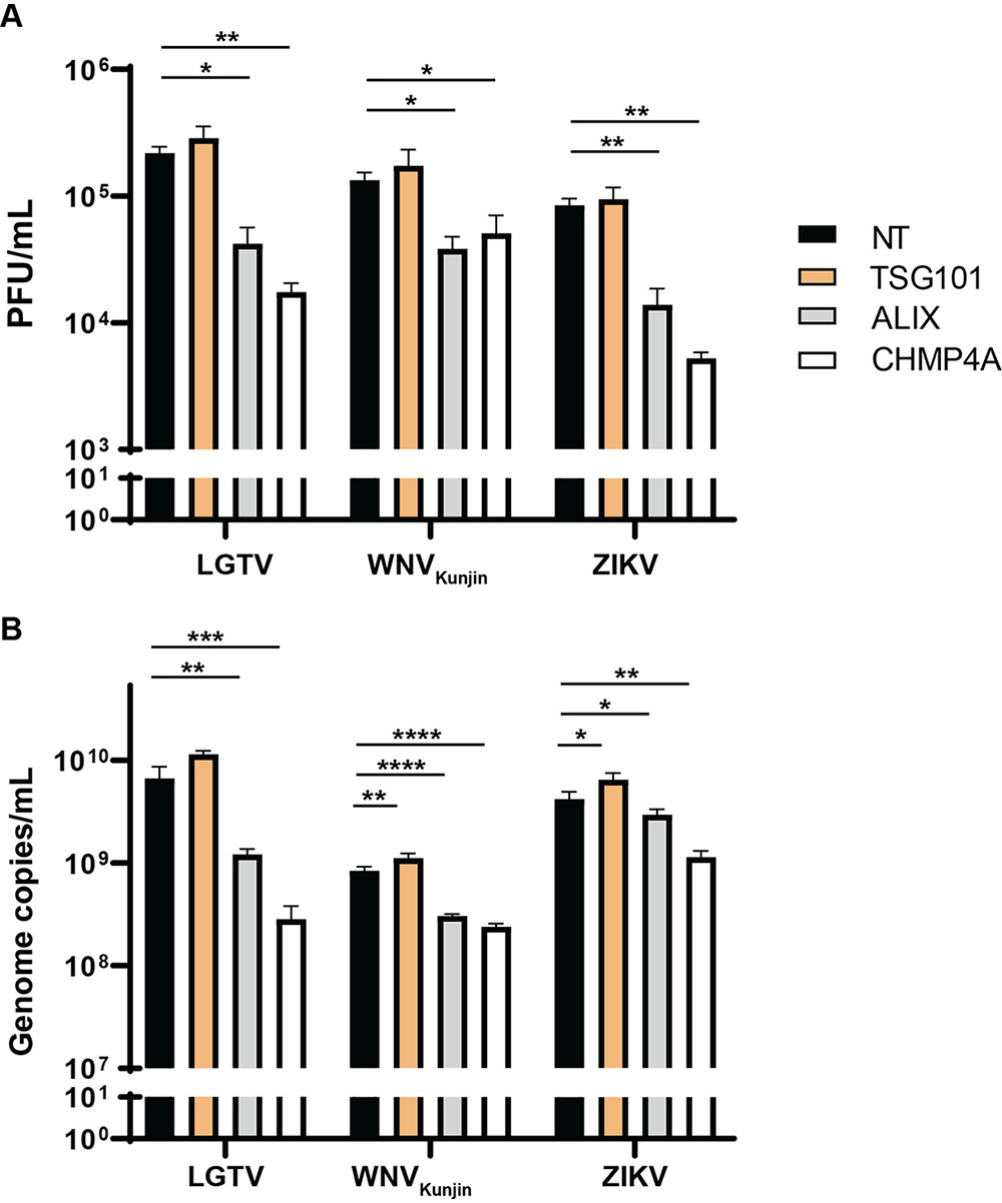
Reduction of intracellular virus particles and virus genome copy numbers during ALIX or CHMP4A depletion. (A) Virus titers in cell lysates from TSG101, ALIX, or CHMP4A siRNA-treated cells were compared to the control NT siRNA-treated cells after LGTV, WNV_Kunjin_, ZIKV infection. The virus titers were measured by plaque assay. (B) Virus genome copy numbers were measured by qPCR from cell lysates of indicated siRNA-treated cells. The experiments were conducted independently three times with two technical repeats. The *P values* are indicated using * *P* < 0.05, ** *P* < 0.01, *** *P* < 0.001, and **** *P* < 0.0001.

As the ESCRT proteins affected virus production, we sought to identify their roles during earlier stages before the virus release. We then determined virus titers and genome copy numbers intracellularly by repeatedly freezing and thawing infected cells. In accordance with virus titers in the supernatants, there was significant reduction of intracellular viruses. Silencing of ALIX or CHMP4A resulted in a 1 log reduction of virus titers, measured by plaque assay (Fig. 2A). Similarly, virus genome copy numbers from cell lysates were reduced when ALIX and CHMP4A were depleted. Particularly, LGTV infected cells showed the highest reduction (1 log) during CHMP4A, compared to the control. This effect was less severe in ZIKV and WNV_Kunjin_ infected cells, with virus production reduced by half of a log (Fig. 2B), which indicated the preferred utility of ALIX and CHMP4A by LGTV. The similarity between results from extracellular and intracellular plaque assays suggests the ESCRT proteins do not act on virus release. The qPCR results from cell lysates suggest ALIX and CHMP4A play a role in either the replication or virus assembly stages.

### Differential requirements of ALIX and CHMP4A in the flavivirus life cycle.

**FIG 3:**
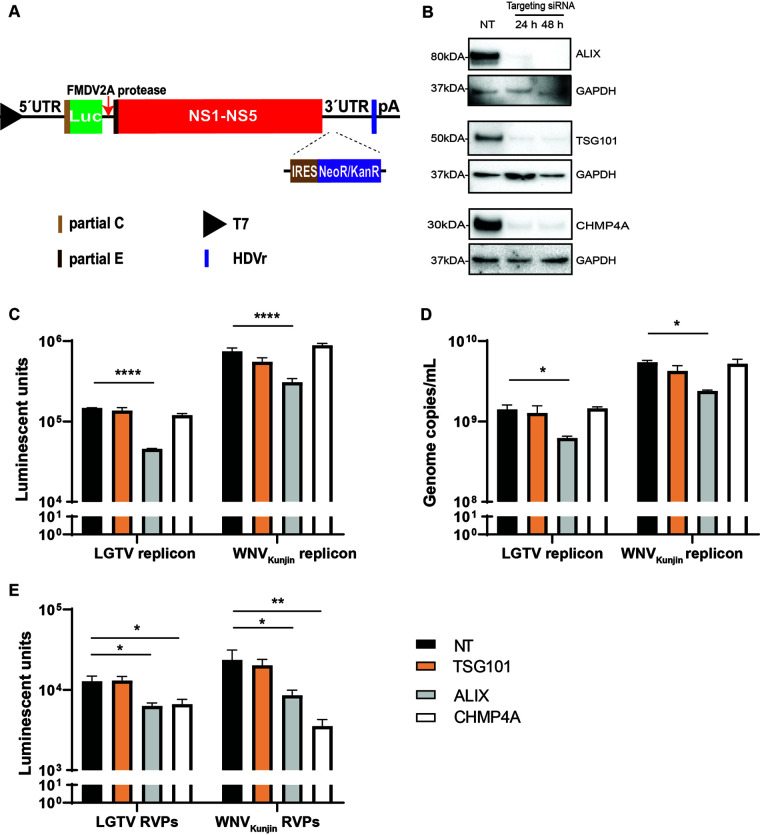
Roles of the ESCRT proteins during flavivirus infection. (A) Schematic illustration of the T7 driven RNA replicon construct comprising: 5′-untranslated region (UTR), the firefly luciferase gene (Luc) as a reporter gene substituted for most genes coding the structural proteins, the foot-and-mouth disease virus autoprotease 2a (FMDV 2A), all the nonstructural proteins, the 3′-UTR, the antigenomic hepatitis delta virus ribozyme (HDVr) sequence, and the simian virus 40 (SV40) polyadenylation signal (pA). An internal ribosome entry site (IRES) sequence and the neomycin/kanamycin resistance (NeoR/KanR) gene were inserted inside the 3′-UTR. (B) Immunoblotting of baby kidney hamster 21 (BHK-21) cell lysates 24–48 h posttransfection with antibodies against either CHMP4A, TSG101, or ALIX after specific siRNA treatments. The expression levels were normalized using the endogenous protein GAPDH. (C) Luminescent units from the BHK21 cell lysates expressing the WNV_Kunjin_ or the LGTV replicons during TSG101, ALIX, or CHMP4A silencing in comparison with the control. (D) Replicon genome copy numbers measured by qPCR from the indicated siRNA-treated cells. (E) Luminescent units from the naive BHK-21 cells infected by the replicon virus-like particles (RVPs) during indicated siRNA treatments compared to the control. The experiments were conducted independently three times with two technical repeats. The *P values* are indicated using * *P* < 0.05, ** *P* < 0.01, and **** *P* < 0.0001.

As we speculated that components of the ESCRT proteins may act during virus replication or assembly, we generated cell lines expressing RNA WNV_Kunjin_ or LGTV replicons to specifically study virus replication. Here, the RNA replicons (Fig. 3A) were transfected into baby kidney hamster 21 (BHK-21) cells. The 3’UTR was engineered with an internal ribosome entry site (IRES) to drive the expression of a neomycin/kanamycin resistance gene IRES-NeoR/KanR, allowing for the selection of cells expressing the subgenomic replicons (28). We also characterized expression of the ESCRT proteins during siRNA treatment of BHK-21 cells. Immunoblotting of cell lysates revealed that gene knockdown resulted in reduction of protein expressions (Fig. 3B).

Depletion of endogenous ALIX resulted in significant reduction in the expression of WNV_Kunjin_ and LGTV replicons, up to 0.5 log, as monitored by the activity of luciferase reporter gene (Fig. 3C) and the genome copy number (Fig. 3D). siRNA knockdown of TSG101 and CHMP4A did not impact the subgenomic RNA replication, a result that reveals a specific role of ALIX in flavivirus replication.

As silencing of CHMP4A did not affect virus replication, we speculated that the protein may have a role in virus assembly. To test this hypothesis, the ESCRT proteins were depleted in cell lines expressing the replicons. This was followed by transfection of the C-prM-E constructs into these cells to supply the structural proteins in *trans*, generating replicon virus-like particles (RVPs). The supernatants containing RVPs were then used to infect naive BHK-21 cells. This was then followed by examining the expression of the luciferase reporter gene. As expected, silencing of CHMP4A resulted in significant reduction in RVPs production (about 0.5 log) (Fig. 3E). There was reduction in luciferase units in RVPs-infected cells during ALIX depletion, which could have resulted from the reduction of replication by ALIX silencing (Fig. 3E). Thus, we conclude that CHMP4A plays a role during virus assembly. These data suggest members of the ESCRT complex perform multiple functions during flavivirus infection.

### ALIX is recruited to the virus replication sites.

**FIG 4:**
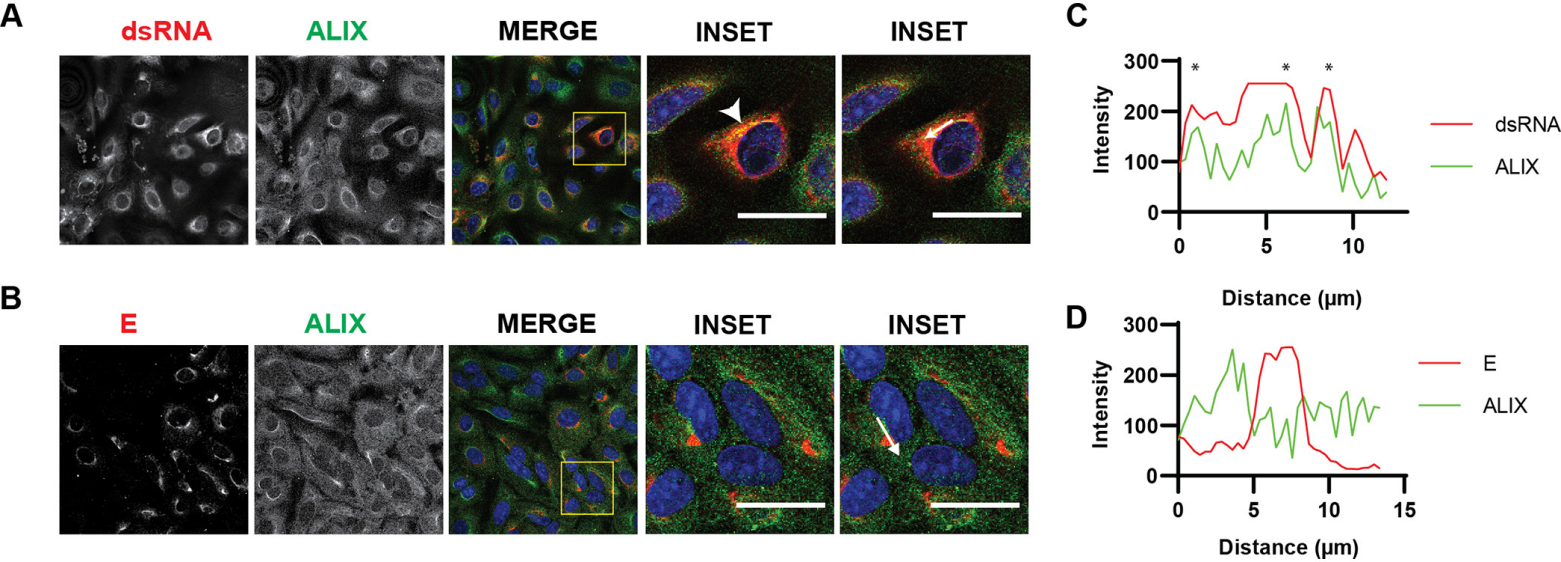
ALIX is recruited to the virus replication sites. Immunostaining of A549 cells 48h after infection with LGTV. (A) The cells were labeled with anti dsRNA (red) and ALIX (green), while in (B) the cells were labeled with anti E (red) and ALIX (green). The nuclei were counterstained with DAPI (blue). The insets represent images from the yellow squares. The arrowhead points to the area where there is high colocalization of red and green fluorophores, resulting in yellow fluorophores. Bar scales represent 20 μm. (C) and (D) Graphs illustrate green and red fluorescent intensity at the arrow in the panel (A) and (B), respectively. Stars indicate the overlapping peaks of fluorescent intensity.

As ALIX has a role during virus replication, we hypothesized that the protein can be recruited to the replication sites. A549 cells infected with LGTV were immuno labeled with antibodies against either dsRNA or E protein together with ALIX. There was a strong overlap between red and green fluorophores during costaining dsRNA and ALIX, suggesting the colocalization of the protein at the replication sites (Fig. 4A and C). There was, however, no colocalization between the ALIX and the E proteins (Fig. 4B and D). Unfortunately, we could not successfully label other ESCRT proteins during the staining. Thus, these data suggest that ALIX was recruited to the replication sites during LGTV infection.

### ESCRT proteins close localize and interact with NS3 and E.

**FIG 5:**
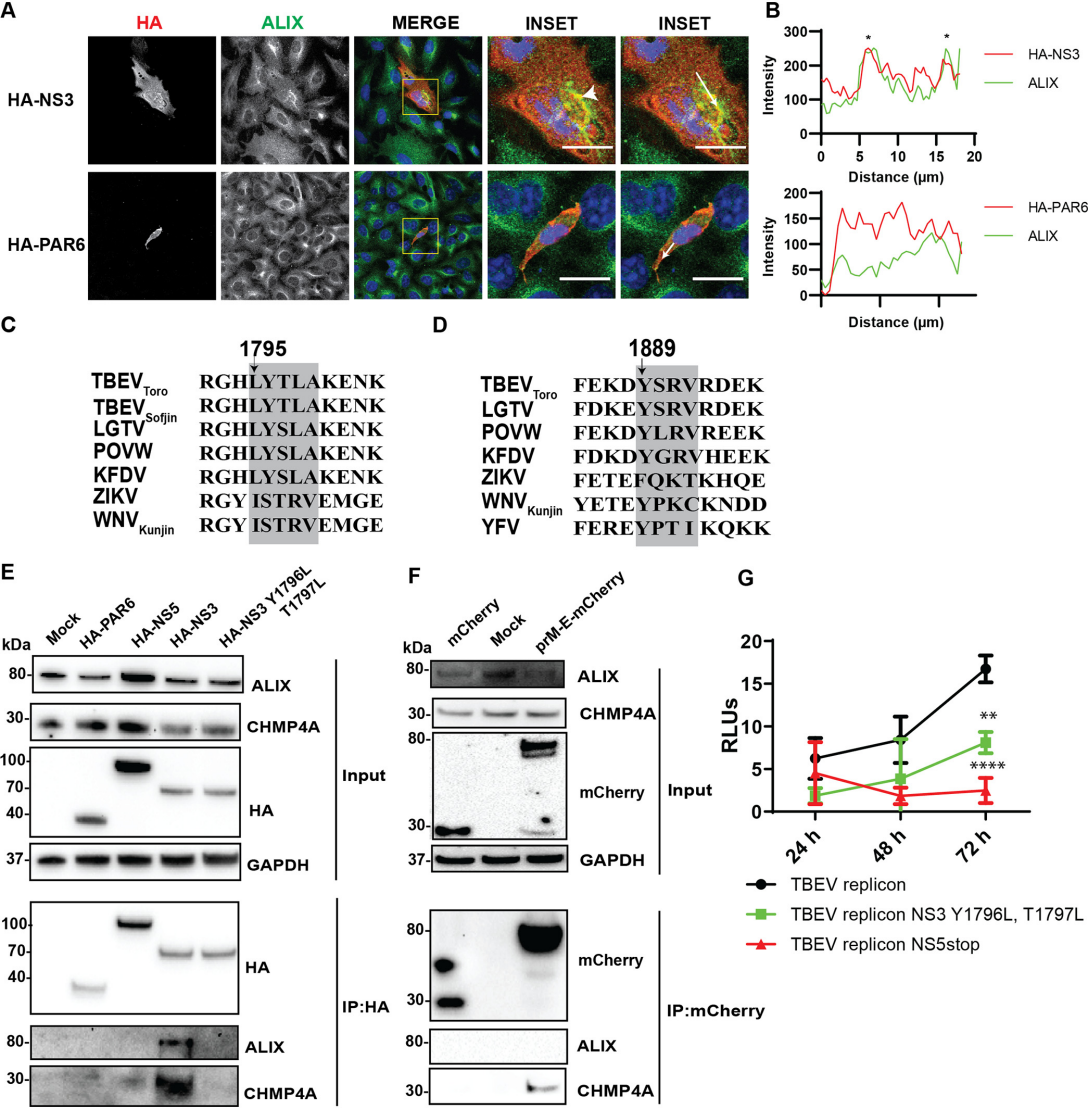
Close localization and interaction between the ESCRT proteins and the TBEV_Toro_ NS3 proteins. (A) Immunostaining of A549 cells transfected with the HA-NS3 or HA-PAR6 construct. The cells were labeled with anti HA (red) and ALIX (green). The nuclei were counterstained with DAPI (blue). The insets represent images from the yellow squares. The arrowhead points to the area where there is high colocalization of red and green fluorophores, resulting in yellow fluorophores. Bar scales represent 20 μm. (B) Graphs illustrate green and red fluorescent intensity at the arrow in the panel (A). Stars indicate the overlapping peaks of the fluorescent intensity. (C) Schema illustrates the conserved late domain motif LYXXA inside the NS3 protein beginning at the 1795^th^ amino acid from the first methionine of the polyprotein, using TBEV_Toro_ as a reference virus. (D) Schema illustrates another putative L domain sequence YPTI within the YFV NS3 protein as described in (24). (E) Immunoblotting of cell lysates 48 h posttransfection with HA-PAR6, HA-NS5, HA-NS3, or HA-NS3 Y1796L T1797L constructs, and the immunoprecipitation (IP) eluates by an anti-HA antibody. The proteins were visualized with the antibodies against HA, ALIX, CHMP4A, and GAPDH as the loading control. (F) Immunoblotting of cell lysates 48 h posttransfection with mCherry, prM-E-mCherry constructs, and the eluates after pulling down by an anti-mCherry antibody. The proteins were visualized with the antibodies against mCherry, ALIX, CHMP4A, and GAPDH as the loading control. (G) Relative luminescent units (RLUs) from the A549 cells transfected with a renilla luciferase-expressing construct and either DNA TBEV_Toro_ replicon, TBEV_Toro_ replicon with Y1796L T1797L mutations, or truncated TBEV_Toro_ replicon with a stop codon inserted in NS5 (35).

Since we demonstrated that ALIX is recruited to the replication site, we investigated interactions of ESCRT components with viral proteins. As the depletion of the ESCRT proteins produces a strong inhibitory effect on LGTV and other closely related viruses in the TBFV serocomplex (29–31), we used LGTV and TBEV_Toro_ gene constructs as models for studying protein-protein interactions. We transfected A549 cells with the construct expressing HA epitope tag-TBEV_Toro_ NS3 and using the construct HA-PAR6 as a control. We show that ALIX colocalizes with NS3 but not with the control (Fig. 5A and B).

We observed an L domain sequence motif LYTLA beginning at the 1795th amino acid of the polyprotein within the TBEV_Toro_ NS3 protein, much like in the retrovirus Gag proteins (32, 33), which may be the putative interaction site between NS3 and the ESCRT proteins (Fig. 5C). We transfected cells with HA-PAR6, HA-NS5 constructs as controls, HA-NS3, and the double mutants HA-NS3 Y1796L T1797L, followed by immunoprecipitations (IP) with an HA antibody to pull down the endogenous ESCRT proteins. After the IP, both ALIX and CHMP4A could be pulled down from the cell lysates expressing HA-NS3 but not from the other controls (Fig. 5E), indicating the interaction between the ESCRT proteins and NS3, and the putative L domain motif were essential for interaction between NS3 and the ESCRT complex.

As there is colocalization between E and CHMP4A during JEV infection (23), we investigated whether there are interactions between the proteins in the context of TBEV. We transfected cells with the TBEV_Toro_ anchored prM-E-mCherry or the mCherry construct, followed by IP with the mCherry antibody. Here, the anchored domain of the capsid protein in the anchored prM-E construct allowed the protein to locate at the ER membrane with its correct topology. During expression, the prM is cleaved from the protein by a host peptidase and results in E-mCherry (34). After the IP, we could pull down CHMP4A but not ALIX (Fig. 5F), suggesting that a direct interaction between CHMP4A and E.

Furthermore, we took advantage of the DNA TBEV_Toro_ replicon having firefly luciferase as a reporter gene, as described previously (35). The replicon transfection can be normalized by cotransfecting the replicon with another construct expressing renilla luciferase. Thus, the replicon expression can be enumerated by the ratio of firefly luminescence to renilla luminescence, presented as relative luminescent units (RLUs). We also generated a DNA TBEV_Toro_ replicon construct having Y1796L and T1797L mutations. Compared to the TBEV_Toro_ replicon, there was significant reduction in the RLUs expressed by the Y1796L and T1797L mutation replicon 72 h after transfections (Fig. 5G). A negative-control replicon with NS5 truncated (35) was used to show that the replicon expressions were not driven by the promoter of the DNA replicon construct. All together, these data suggest that the TBEV NS3 interacts with the ESCRT proteins at the putative late domain that supports virus replication and the E protein interact only with CHMP4A.

### Expression of ISG15/HERC5 resulted in reduction in LGTV replication and enhancement of the ALIX-depleting effect.

**FIG 6:**
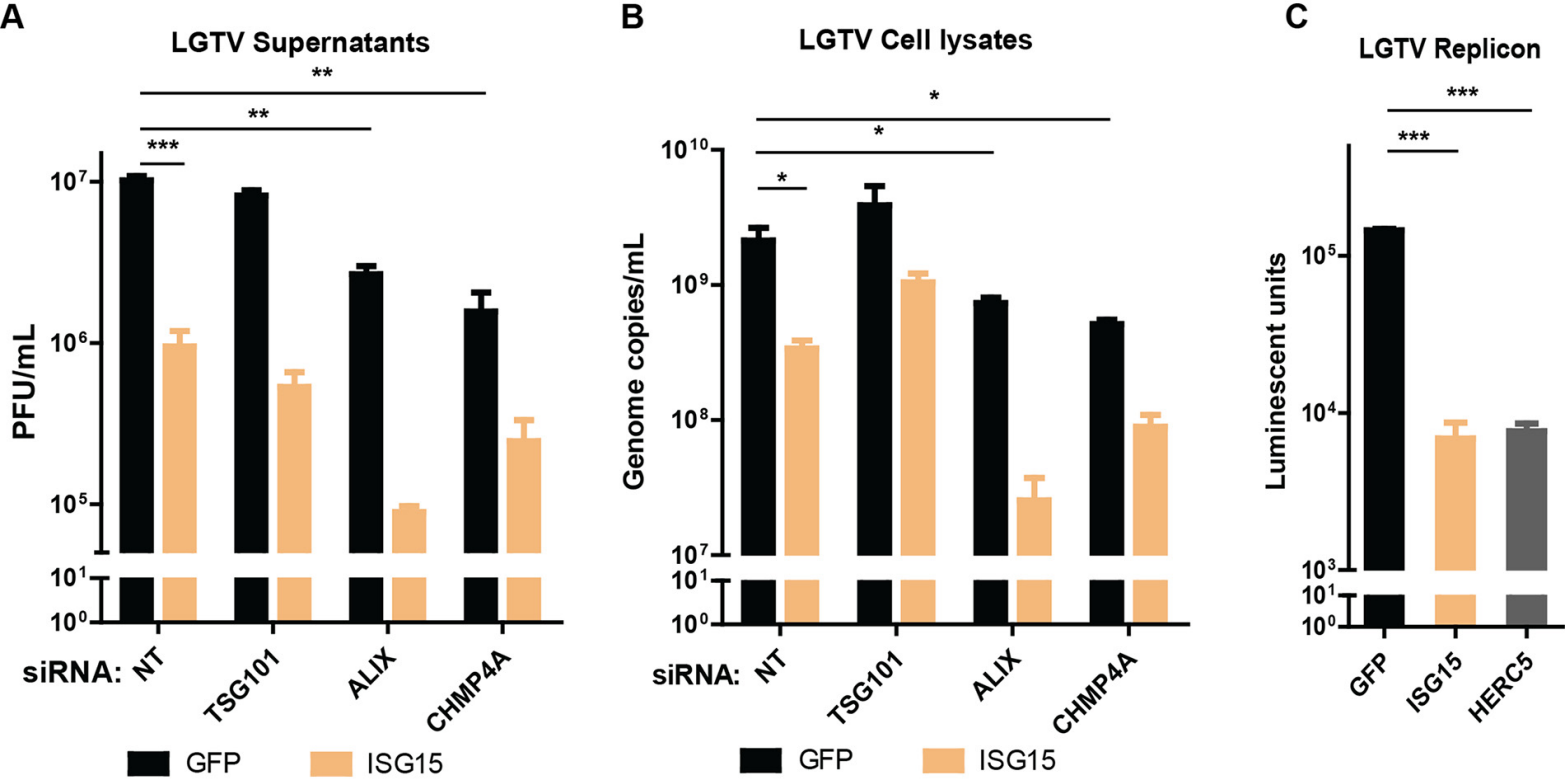
ISG15 expression enhanced effects of ALIX or CHMP4A depletion. (A) LGTV titers from supernatants of A549 cells transfected with either ISG15 or GFP constructs and with indicated siRNA, measured by plaque assay. (B) LGTV genome copy numbers from lysates of A549 transfected with indicated constructs and siRNA, measured by qPCR. (C) Expression of luciferase reporter from the BHK-21 cell line expressing the LGTV replicon and transfected with either GFP, ISG15 or HERC5 constructs. The experiments were conducted independently three times with two technical repeats. The *P values* are indicated using * *P* < 0.05, ** *P* < 0.01, and *** *P* < 0.001.

As TSG101 can be ISGylated during IAV infection to reduce the virus budding (20), we investigated the similar interactions between ISG15 and the ESCRT proteins during TBFV infection. A549 cells were treated with ESCRT siRNA and transfected with either GFP as a control or ISG15 construct before LGTV infection. Expression of ISG15 resulted in a 1 log reduction in LGTV titer, compared to the control (Fig. 6A). Remarkably, expression of ISG15 concomitantly with depletion of ALIX synergistically reduced the virus titers (Fig. 6A), compared to that of TSG101. In accordance with virus titers from cell culture supernatants, there was an enhanced 2 log reduction of intracellular virus genome copy number during ALIX depletion and ISG15 expression (Fig. 6B), suggesting ISG15 directly targets ALIX. Furthermore, on the replicon platform, expression of either ISG15 or HERC5 resulted in more than 1 log reduction of luciferase units (Fig. 6C), suggesting their restricting effects on virus replication. These data suggest that ISG15 and HERC5 expression reduce virus replication, and ISG15 may interplay with ALIX during virus infection.

### Unconjugated ISG15 and HERC5 elicited ALIX and CHMP4A degradation.

**FIG 7:**
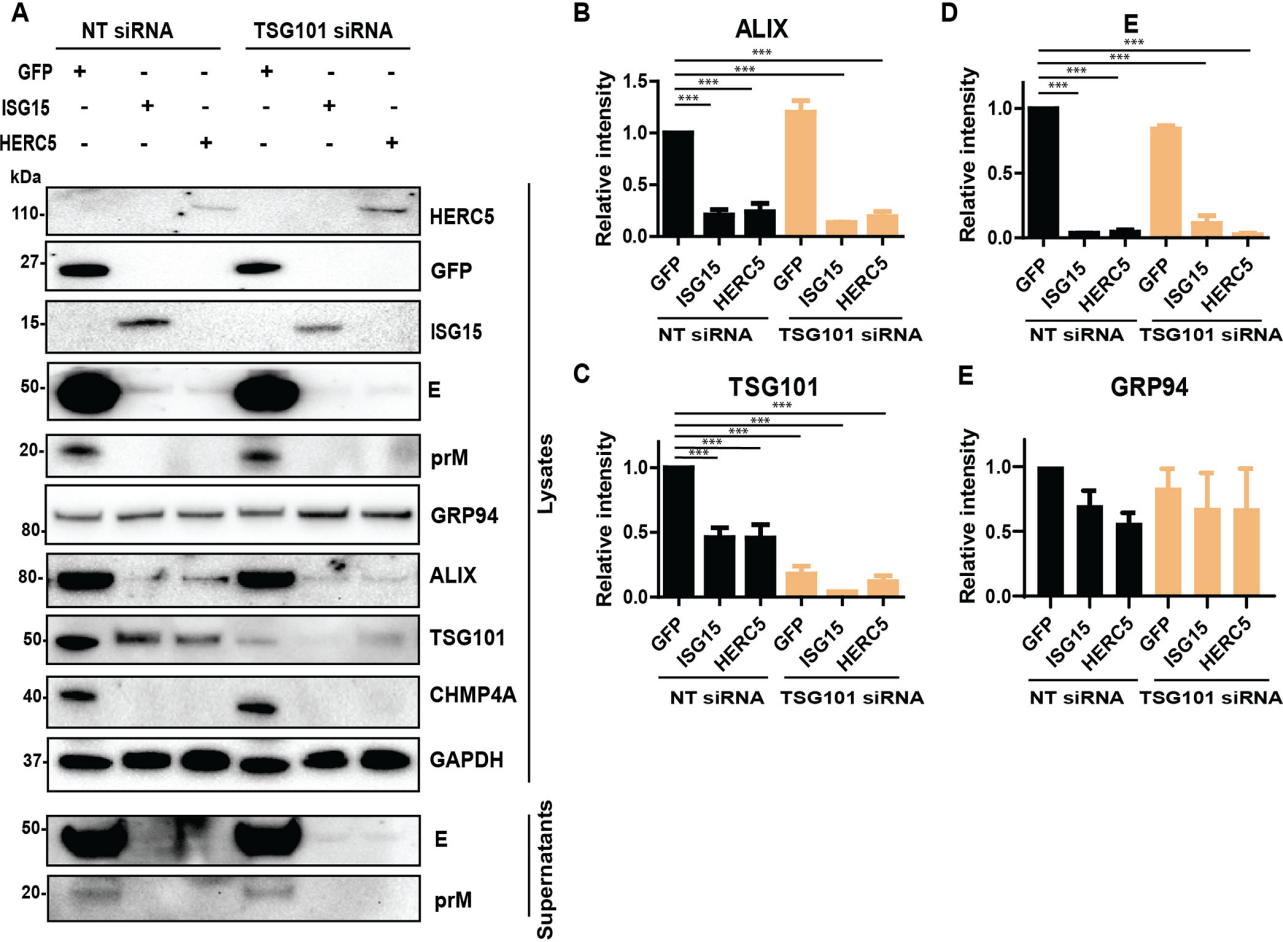
Expression of ISG15 and HERC5 resulted in degradation of TSG101, ALIX, and CHMP4A. (A) Immunoblotting of supernatants and cell lysates transfected with either GFP, ISG15, or HERC5 constructs and treated with either NT or TSG101 siRNA. The cells were then infected with LGTV. The proteins were visualized with the antibodies against GFP, ISG15, HERC5, TSG101, ALIX, CHMP4A, the 94 kDa glucose-regulated protein (GRP94), E, prM, and GAPDH as the loading control. (B), (C), (D), and (E) Relative intensity of the ALIX, TSG101, or GRP94 protein bands from the blots in (A). The experiments were conducted independently three times. The *P values* are indicated using *** *P* < 0.001.

As the expression of ISG15 enhances the effects of the ESCRT protein depletion during flavivirus infection and TSG101 can be targeted by ISG15 during IAV infection (20), we investigated the effects of ISG15 on the ESCRT proteins during flavivirus infection. A549 cells were cotransfected with the siRNA targeting TSG101 and constructs expressing either ISG15, HERC5, or the control GFP before LGTV infection (Fig. 7A). As expected, expression of ISG15 or HERC5 reduced generation of virus particles compared to the control, whereas silencing of TSG101 did not alter virus production in both cell lysates and supernatants, indicated by immunoblotting of extracellular E and prM (Fig. 7A and D). Interestingly, expression of ISG15 or HERC5 resulted in 50% degradation of TSG101 (Fig. 7A and C) and much stronger degradations of ALIX and CHMP4A: 80% of ALIX was degraded (Fig. 7A and B), and CHMP4A was not detectable during ISG15 and HERC5 expression (Fig. 7A). However, we did not observe alterations in level of the 94-kDa glucose-regulated protein (GRP94) (Fig. 7A and E), which is an ER marker protein (36), suggesting the specific ALIX and CHMP4A degradation by ISG15 or HERC5 expression. Furthermore, we could not detect signs of ISGylation on the blotting membranes as the ISG15 was expressed without the enzymes required for the ISGylation pathway (26, 27, 37–39). Altogether, these results demonstrate that expression of unconjugated ISG15 or HERC5 degraded ESCRT proteins during LGTV infection, which may explain the enhanced virus inhibition during concomitant expression of ISG15 and depletion of ALIX.

## DISCUSSION

During DENV and JEV infection, TSG101, CHMP2, and CHMP4 play roles in virus assembly (32). Here, we show that silencing of ALIX and CHMP4A reduced titers of the TBFV, including LGTV, KFDV, POWV, and TBEV_Sofijn_, similar to MBFV WNV_Kunjin_ and ZIKV (Fig. 1). We found that ALIX is recruited to the replication sites (Fig. 4), supporting virus replication, and CHMP4A is essential for virus assembly (Fig. 3).

**FIG 8:**
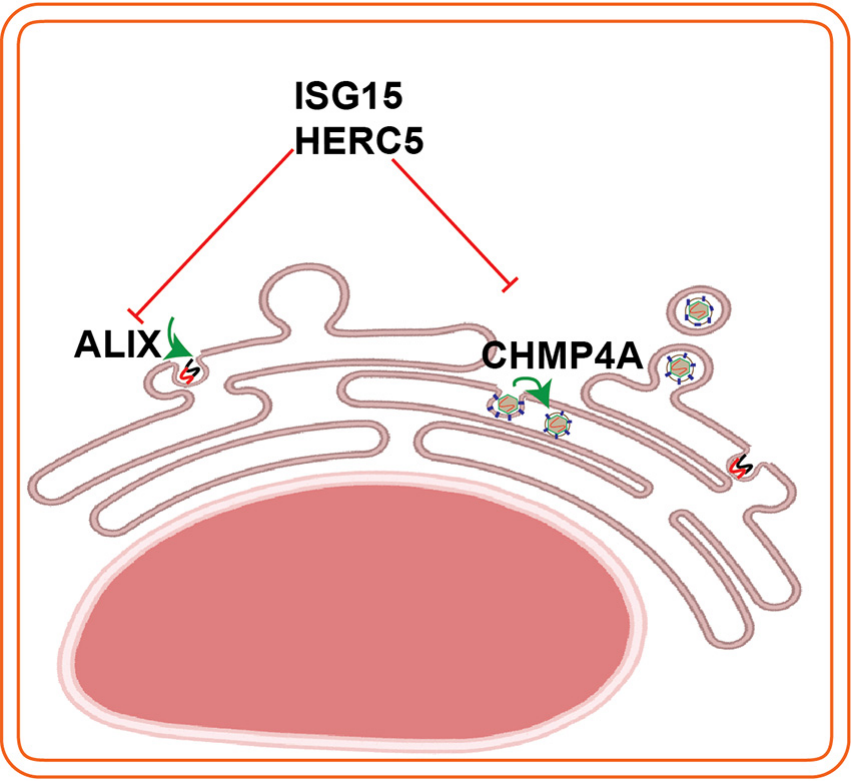
Schema illustrates interplays between the ESCRT proteins (ALIX, CHMP4A) and the ISG15/HERC5. ALIX has roles in virus replication and CHMP4A is required for virus assembly. To counteract virus infection, infected cells induce the ISG15/HERC5 system to degrade the ESCRT proteins. The schema was created using Biorender web tool.

Contrary to many other enveloped viruses, where the assembly occurs at the plasma membrane, assembly in flaviviruses is coupled to replication at the ER (6). At the replication site, ALIX-CHMP4A form a complex with NS3 as NS3 can pull down the proteins (Fig. 5). A crystal structural study has revealed that the ALIX protein has two domains: Bro1 and V (40). Studies on retroviruses have shown that ALIX can mutually interact with other ESCRT proteins and the virus proteins; indeed, ALIX interacts with the L domain motif within the retrovirus p6 protein at its V domain (41) and the protein interacts with CHMP4A at its Bro1 domain (42). At the assembly site, CHMP4A is recruited to E, supporting virus assembly as we could pull down the CHMP4A by the E protein. Correlative light electron microscopy has been shown that CHMP4A colocalize at both the replication and virus assembly sites during JEV infection (23). Therefore, we propose a model for the roles of ALIX and CHMP4A at replication and assembly site, respectively, during TBFV infection (Fig. 8).

We identified the LYTLA motif within the helicase domain of the TBEV_Toro_ NS3 protein. The sequence is similar to the L domain motif ФYXФXL recruiting ALIX described in retroviruses (Ф being any nonpolar amino acid) (19, 43). The identified motif is highly conserved in the TBFV with the polar YT/S residues flanked by nonpolar residues (Fig. 5C). Based on this observation, we mutated the YT residues, which aborted the interaction between NS3 and the ESCRT complex (Fig. 5E). However, the equivalent ISTRV sequence detected within NS3 of the MBFV is less similar to the putative TBFV L domain motif in this study. Thus, MBFV may have other L motifs within NS3. Indeed, there is another proposed putative L domain motif within YFV NS3 protein supporting ALIX recruitment (Fig. 5D) (24).

TSG101 assists post Golgi transporting of IAV HA to the plasma membrane (20). In our study, the effect of TSG101 seems dispensable as their depletions did not highly alter virus production. These findings on TSG101 differ from a previous study showing TSG101 is required for JEV and DENV assembly, although interestingly ALIX appeared to be dispensable (23). However, our findings are consistent with another study on YFV that showed that ALIX is essential for the virus infection (24). These findings suggest that there are variances in the use of either ALIX or TSG101, which are the two early docking proteins of the ESCRT complex to the virus proteins (18, 32).

Lastly, we identified that unconjugated ISG15 and HERC5 inhibit LGTV replication, and their expression enhanced the effects of ALIX silencing (Fig. 6) due to the degradation of the ESCRT complex (Fig. 7). We hypothesize that the degradation can be dependent on lysosome as shown in a previous study (44). Furthermore, we do not eliminate the effects of ISG15 on other proteins as ISG15 has a broad spectrum of targeting proteins (45, 46). In addition, the ISGylation process can be induced by the interferon response or expressing ISG15 concomitantly with the key enzymes of ISGylation steps (45, 46). Although ISGylation has been shown during DENV infection (47), we could not identify any signs of ISGylation during LGTV infection within A549 cells. Thus, to our knowledge, this is the first time it has been shown that the unconjugated ISG15 and HERC5 can inhibit LGTV replication and trigger ESCRT protein degradation. Similarly, it has been shown that ISG15 or HERC5 can inhibit chikungunya (48) and HIV-1 (49), respectively, independently of ISGylation. However, our new findings with ISG15 and HERC5 do not exclude the effects of ISG15 on other stages of the flavivirus life cycle as the recombinant ISG15 has been shown to interfere with ZIKV entry (50) and DENV release (51).

In summary, we identified and characterized checkpoints such as the interaction between ESCRT complex–NS3 and the interplay between ISG15–ESCRT complex, which can function as future targets for the development of antiviral therapies. Future studies should be performed to investigate whether the virus protease NS2B/NS3 can cleave ISG15 to antagonize its antiviral function. Our study has provided insights into virus-host cell interactions important for the flavivirus life cycle.

## MATERIALS AND METHODS

### Cell culture.

BHK-21 (ATCC), Vero (ATCC), and A549 (ATCC) cells were maintained in Dulbecco’s Modified Eagle’s Medium (DMEM) containing 1 g/liter glucose (Gibco) supplemented with 10% heat-inactivated fetal bovine serum (HI–FBS) (Gibco) and 100 U/ml penicillin-streptomycin (PEST) (Gibco) at 37°C in 5% CO_2_.

### Gene constructs.

The WNV_Kunjin_ replicon was constructed based on the WNV_Kunjin_ sequence (accession number AY274504), and the LGTV replicon was constructed based on the LGTV strain TP21 sequence (accession number NC003690). The constructs are driven by a T7 promoter for *in vitro* transcription to express the replicons as described in (28) (Fig. 3A).

The TBEV NS3 and NS5 (Torö-2003, AH013799) genes were amplified by PCR with primers introducing suitable endonuclease restriction sites. Full-length NS5 and NS3 were cloned into the mammalian cell expression pKH3, which were kindly provided by Ian Macara and Ben Margolis, to have a triple HA tag at the N terminus. NS3 mutations were generated by PCR of the HA-NS3 construct or the replicon construct (35) with 5′ phosphorylated primers introducing mutated sites, followed by a ligation. The product was treated with restriction enzyme DpnI (Thermo Scientific) to remove the methylated plasmid PCR template.

The TBEV_Toro_ anchored prM-E (Torö-2003, AH013799) genes were amplified by PCR with primers introducing suitable endonuclease restriction sites. Full-length anchored prM-E was cloned into the mammalian cell expression pmCherry N1 (Clontech) with the mCherry tag at the C terminus.

Renilla luciferase expressing construct (pGL4.74, Promega) was used for normalizing transfections of TBEV_Toro_ DNA replicons.

### siRNA transfection.

70% confluent A549 cells in 24-well plate were transfected with 10 pmol of SMART pool siRNAs specific against TSG101 (Catalogue number: L-003549, Horizon Discovery), ALIX (Catalogue number: L-004233, Horizon Discovery), CHMP4A (Catalogue number: L-020698, Horizon Discovery), or nontargeting (NT) siRNA (Catalogue number: D-001810-01-20, Horizon Discovery), using lipofectamine RNAiMAX reagent (Invitrogen) for 24 h.

### Cytotoxicity assay.

Cell lysates and media were assayed using the CyQUANT LDH Cytotoxicity Assay (Invitrogen). The absorbance was read at 490 nm and normalized to the absorbance at 680 nm using the Cytation 3 Multi-Mode Reader (BioTek, Bad Friedrichshall, Germany).

### Virus infection and cell harvesting.

After siRNA treatments for 24 h, A549 cells were infected by TBEV_Sofjin_, KFDV, POWV, WNV_Kunjin_, ZIKV, or LGTV with 0.1 multiplicity of infection. After 24 h, supernatants were harvested. Attached cells were detached by trypsinization, followed by a soybean trypsin inhibitor treatment (Gibco). Cells were then briefly frozen using liquid nitrogen and thawed; this was done three times.

### Antibodies.

The following antibodies were used in this study: mouse monoclonal anti-TBEV E (United States Army Medical Research, Institute of Infectious Diseases, Fort Detrick, Frederick, MD, USA), mouse monoclonal anti-TBEV prM (United States Army Medical Research), mouse monoclonal anti-TSG101 (Santa Cruz), mouse J2 anti-dsRNA (SCICONS, Hungary), mouse monoclonal anti-ALIX (Santa Cruz), mouse monoclonal anti-CHMP4A (Santa Cruz), rabbit anti-TSG101 (Atlas antibodies), rabbit anti-CHMP4A (Invitrogen), rabbit anti-ALIX (Atlas antibodies), rabbit anti-HA tag (Invitrogen), mouse anti-HA tag (Invitrogen), rapid anti-GRP94 (Invitrogen), mouse anti-GAPDH (Sigma), mouse anti-βactin (Sigma), mouse monoclonal anti-mCherry tag (Novus Biological), rabbit monoclonal anti-mCherry tag (Novus Biological), Alexa Fluor 594-conjugated anti-mouse goat antibody (Invitrogen), Alexa Fluor 488-conjugated anti-rabbit goat antibody (Invitrogen), anti-mouse VisUCyte HRP Polymer (R&D Systems, USA), and HPR-conjugated anti-mouse goat antibody (Invitrogen).

### Plaque assay.

Crystal violet-based plaque assay was performed to quantify WNV_Kunjin_ and ZIKV, and immunofocus plaque assay was performed to quantify LGTV, TBEV_Sojin_, KFDV, and POWV. In brief, a series of virus dilutions in DMEM were used to infect 90% confluent Vero cells for 1 h at 37°C, followed by cell-overlaying with DMEM supplemented with 1.2% Avicel (FMC), 2% HI–FBS (Gibco), 1× nonessential amino acids (Gibco), and 1% PEST (Gibco). After 3–5 days, the overlays were removed, and cells were fixed by methanol for 20 min before staining. For immunofocus assay, the fixed cells were blocked by 2% BSA (Fitzgerald) for 10 min before labeled with 1:1000 the mouse anti-E antibody, followed by 1:100 the anti-mouse secondary HRP Polymer for 1 h at 37°C. Finally, they were reacted with KPL TrueBlue Peroxidase Substrate (Seracare) for 15 min at room temperature (RT). For crystal violet-based plaque assay, the fixed cells were stained with 2% crystal violet (Sigma), 20% methanol (Fisher), and 0.1% ammonium oxalate (Sigma) solution.

### Quantitative real-time PCR.

Total RNA was isolated from cell lysates or cell culture supernatants using RNeasy minikit (Qiagen) or QIAamp Viral RNA minikit (Qiagen), respectively. cDNA was synthesized using High-Capacity cDNA Reverse Transcription kit (Applied Biosystems) with specific primers targeting the WNV_KUN_, ZIKV, or LGTV positive strand (Table 1). qPCR was conducted using a QuantStudio 7 Flex real-time PCR system (Applied Biosystems) with TaqMan Fast Advanced Master Mix (Applied Biosystems) and Custom TaqMan Gene Expression Assays (Applied Biosystems) containing specific primers and probes (Table 1). Virus genome copy numbers were enumerated by extrapolation from the standard curves of Ct values and known genome copy number solutions.

**TABLE 1:** List of primers and probes used for qPCR

Assay	Virus	Sequence (5′–3′)
cDNA synthesis	WNV_Kunjin_	AATATGCTGTGTTGTTGTGG
	ZIKV	GATCTTGGTGAATGTGAACG
	LGTV	CTCCCTGTGAGTTCATAATTGG
qPCR assay	WNV_Kunjin_	CAGACCACGCCATGGCG
		CTAGGGCCGCGTGGG
		FAM-TCTGCGGAGAGTGCAGTCTGCGA-NFQ
	ZIKV	CCGCTGCCCAACACAAG
		CCACTAACGTTCTTTTGCAGACAT
		FAM-AGCCTACCTTGACAAGCAGTCAGACACTCAA-NFQ
	LGTV	ACTGAACTGGAGAAGGAGGA
		CCACAGTCCCATGACGATAAG
		FAM-TAGGCTTGATTGCCTCGGCCTTTC-NFQ

### Luciferase assay.

Cell lysates were assayed and measured for bioluminescence using Dual-Luciferase Reporter assay kit (Promega) and a Lumi-star Omega machine (BMG Labtech), according to the manufacturer’s instructions.

### Protein electrophoresis and immunoblotting.

Cells were lyzed with 0.5% SDS before boiling in LDS sample buffer (Invitrogen). Proteins were separated on precast 4%–12% polyacrylamide Bis-Tris gels (Invitrogen) in MES running buffer (Invitrogen) for 1 h at 120 V constant before transferred to nitrocellulose membranes using an iBlot 2 Gel Transfer device (Invitrogen). Proteins of interest were detected with the antibodies.

### Immunoprecipitation.

For pull-down studies, A549 cells were transfected with individual constructs using a Nucleofector 2b device (Lonza) and Cell Line Nucleofector kit T (Lonza). Two days posttransfection, the cells were lyzed by radioimmunoprecipitation assay (RIPA) buffer (Sigma) supplemented with a protease inhibitor cocktail (Sigma) for 20 min at 4°C. Lysates were precleared by centrifuge at 1000g for 10 min, and 300 μg of the precleared lysates were incubated with 4 μg of antibodies and 50 μl of a protein A/G-tagged microbead premix (MACS) at 4°C for 1 h. The mixtures were then loaded on μ Columns (MACS) holding a μMACS Separator to retain the microbeads on the columns. The beads were washed four times with 4°C 50% RIPA buffer. Finally, bead-binding proteins were eluted by a preheated 95°C LDS sample buffer.

### Immunofluorescence labeling.

Cells grown on coverslips were fixed by 4% paraformaldehyde (Scharlau, Spain) for 20 min. They were then permeabilized by 0.1% Triton X-100 (VWR) before being blocked with 2% bovine serum albumin (BSA) (Fitzgerald) and 2% goat serum (Invitrogen) for 1 h. Cells were labeled with the primary antibodies for 2 h at 37°C, followed by the secondary antibodies Alexa Fluor 488 or Alexa Fluor 594. The cells were counterstained with DAPI (Sigma) for 5 min before mounting with ProLong Gold Antifade Mountant (Life Technologies). Images were captured using a confocal laser scanning microscopy SP8 (Leica) and analyzed using ImageJ.

### Protein sequence and alignment.

Polyprotein sequences were assessed from NCBI with the accession number ABD62793.2, AEP20480, NP_620108, AFF18436.1, NP_620099, QOF88708.1, AOS89766.1, NP_041726.1 for TBEV_Toro_, TBEV_Sofjin_, LGTV, POWV, KFDV, ZIKV, WNV_Kunjin_, and YFV. The alignments were conducted using CLUSTERX2.

### Statistical analysis.

Statistical differences between the means were determined using one-way ANOVA followed by the Bonferroni *post hoc* test, and *P* < 0.05 was considered statistically significant difference. GraphPad Prism 9 was used for performing all statistical analyses. The values are presented as mean ± standard error of the mean.

### Data availability.

The data presented in this study are available in this article. Remaining data supporting reported results are available from the corresponding authors upon reasonable requests.
